# Response: Behind the closed doors of mentalizing. A commentary on “Another step closer to measuring the ghosts in the nursery: preliminary validation of the Trauma Reflective Functioning Scale”

**DOI:** 10.3389/fpsyg.2015.00697

**Published:** 2015-06-05

**Authors:** Karin Ensink, Peter Fonagy, Nicolas Berthelot, Lina Normandin, Odette Bernazzani

**Affiliations:** ^1^Université LavalQuébec, QC, Canada; ^2^Research Department of Clinical, Educational and Health Psychology, University College London, Anna Freud CentreLondon, UK; ^3^Université du Québec à Trois-RivièresTrois-Rivières, QC, Canada; ^4^Université de MontréalMontréal, QC, Canada

**Keywords:** mentalization, reflective functioning, dissociation, trauma, abuse

In this thought-provoking commentary on our findings regarding the dramatic deficits in mentalization specifically regarding past experiences of abuse and neglect in adults who otherwise demonstrate that they have the capacity to think about past relationships in terms of affects and mental states despite these histories, Schimmenti re-examines our findings from the perspective of dissociation and subtly raises the question of whether we adequately consider dissociative processes in the interpretation of our findings.

Starting with Ferenzi's early contribution in this regard, Schimmenti points out that dissociative processes are central in reactions to trauma. But what is the relationship between mentalization and dissociation, and more specifically, what is the relationship between mentalization of trauma experiences and dissociation? Recent theoretical and empirical observations suggest that dissociative processes involve a disruption of the normal coordination of affective, memory and cognitive systems, so that unintegrated parallel systems can apparently emerge, with experience being compartmentalized and identity disrupted (Cardeña and Carlson, 2011). Initially this separation functions to keep traumatic memories and affects from disrupting functioning (Foa and Hearst-Ideka, 1996). However, when in the long run this contributes to the experience of the self as fragmented, it becomes highly dysfunctional. As Schimmenti observes, this is especially problematic when it contributes to the continued isolation of a tortured part of the self, namely the affective part of the self that is linked to the abuse.

We find it particularly useful to think about the relationship between mentalizing and dissociation from a developmental perspective, and from what we are learning from our research with sexually abused children. From a developmental perspective, children would have developed a certain level of mentalization, linked to parental reflective functioning, prior to the experience of trauma such as sexual abuse. We consider that mentalization is central to developing a sense of self and identity and that this sense of self will be an important resilience factor in the context of trauma. When the child can depend on support from parents who are able to consider their psychological experience in the context of trauma this may help them to re-establish self-regulation and trust in others, and reduce recourse to dissociative defenses (Ensink et al., unpublished). However, other individual factors such as temperamentally based variations in the predisposition to hypnotisability (Putnam, 1996) may contribute to differential susceptibility to maintaining dissociative reactions once there is no imminent danger, (Lanius et al., 2006). Not surprisingly, children seem to be particularly vulnerable to dissociation (Diseth, 2005; Jans et al., 2008). While dissociation may initially have been used only to avoid trauma related affects or memories are triggered, children may spend more and more time in a state that is disconnected from reality, and may slip into a parallel world either of phantasy, or where thoughts and feeling are suspended. We have previously described this as a relatively typical developmental phase where pretend does not yet have its quality of partial disconnection from reality, but rather is completely severed from the constraints of the “serious” or “real” (Fonagy et al., 2002). From a more clinical perspective, in the context of therapy with sexually abused children, we observe moments of dissociation where their play is interrupted and suspended, apparently without any abuse related triggers, or even other triggers. It is possible that an emotion was activated that in turn disrupted the motivational system and attentional system. This is consistent with the convergent conclusions by a number of authors that dissociation is an exceedingly complex and multi-faceted phenomenon (Briere et al., 2005) so that any particular research operationalization only ever captures a partial glance.

Returning more specifically to our findings with adults who have experienced abuse and neglect, we consider that presently, in the coding and definition of unresolved trauma, dissociative processes are central. Hesse and Main (2000) explicitly make reference to this and describe the momentarily disruptions in cognition and speech production, where in talking about a traumatic experience someone suddenly stops mid-phrase apparently losing the capacity to coordinate and continue speech production or manifest disorientation of person, time and place under the stress of the effort to retrieve trauma related memories. Somewhat surprisingly, in our sample, unresolved trauma, which can be seen as reflecting the potential for dissociation, was not associated with trauma RF. Furthermore, when we examined the implications of trauma RF and unresolved trauma in mothers for infant attachment organization, we have found that both make independent contributions to infant attachment organization (Berthelot et al., 2015). In addition, when we have examined the relationship between RF and dissociation in sexually abused children and adults from the community, we have found that there is a weak to moderate correlation between general RF or mentalization capacities about self and others and scores on questionnaire measures of dissociation (Ensink et al., unpublished). Put differently, what these findings may be suggesting is that when asked to think about trauma, someone may dissociate when overwhelmed by un-metabolized trauma related memories and affects, while also attempting to mentalize. Mentalizing and dissociative processes may thus occur simultaneously in parallel and may be only loosely connected. Interestingly, findings by Lanius et al. (2010) indicate that dissociative responses like detachment, subjective distancing, depersonalization and derealization associated with overmodulation of affect in response to autobiographical trauma memories are mediated by increased medial prefrontal and rostral anterior cingulate activation and decreased amygdala and right anterior insula activation. Considering that mentalization as we are measuring it, involves coordination by prefrontal and temporo-parietal systems (Fonagy and Luyten, 2009), this suggests that closely related neurobiological system are involved in the two processes. If there are different, though to some extend interacting, developmental pathways to mentalization and dissociation, this may explain why we do not see the association between unresolved trauma and trauma RF as we may have intuitively expected. However, we expect that when mentalization is supported in the context of relationships of trust, such as therapeutic relationships, mentalization may be used to become aware of dissociative experiences, metabolize them to some extend and make them more tolerable.

Until this is studied longitudinally we can only speculate regarding the direction of the relationships, but we hypothesize that children with better mentalization capacities may be less likely to dissociate. At the same time, pervasive dissociation when children are not connected to reality and miss out on opportunities to engage in relationship where they can learn about their own minds and that of others, can also be expected to undermine the development of mentalization capacities. However, before coming to the conclusion that dissociation is *necessarily* bad, it is possible that at low levels, dissociation can have adaptive value in everyday life and can actually support emotional regulation and creativity by inhibiting thinking about disturbing experiences (Butler, 2006).

We hope that our response to this commentary will stimulate further reflection and research into dissociation and mentalization and risk and resilience in the context of abuse.

## Conflict of interest statement

The authors declare that the research was conducted in the absence of any commercial or financial relationships that could be construed as a potential conflict of interest.
